# Association of *Matrix Metalloproteinase-9* (*MMP9*) Variants with Primary Angle Closure and Primary Angle Closure Glaucoma

**DOI:** 10.1371/journal.pone.0157093

**Published:** 2016-06-07

**Authors:** Xueli Chen, Yuhong Chen, Janey L. Wiggs, Louis R. Pasquale, Xinghuai Sun, Bao Jian Fan

**Affiliations:** 1 Department of Ophthalmology & Vision Science, Eye & Ear Nose Throat Hospital, Shanghai Medical College, Fudan University, Shanghai, China; 2 Department of Ophthalmology, Harvard Medical School, Massachusetts Eye and Ear Infirmary, Boston, Massachusetts, United States of America; 3 Channing Division of Network Medicine, Brigham and Women’s Hospital, Harvard Medical School, Boston, Massachusetts, United States of America; 4 State Key Laboratory of Medical Neurobiology, Institutes of Brain Science and Collaborative Innovation Center for Brain Science, Fudan University, Shanghai, China; 5 Key Laboratory of Myopia, Ministry of Health (Fudan University), and Shanghai Key Laboratory of Visual Impairment and Restoration (Fudan University), Shanghai, China; Casey Eye Institute, UNITED STATES

## Abstract

Shorter axial length observed in patients with primary angle closure glaucoma (PACG) might be due to altered matrix metalloproteinase-9 (MMP9) activity resulting in ECM remodeling during eye growth and development. This study aimed to evaluate common variants in *MMP9* for association with PACG. Six tag SNPs of *MMP9* were genotyped in a Chinese sample of 1,030 cases, including 572 PACG and 458 primary angle closure (PAC), and 499 controls. None of 6 SNPs were significantly associated with overall PAC/PACG (*P* > 0.07) or with PAC/PACG subgroups (*P*_*c*_ > 0.18). Meta-analysis of two non-Chinese studies revealed significant association between rs17576 and PACG (OR_s_ = 0.56, *P* < 0.0001); however, meta-analysis of our dataset with 4 Chinese datasets did not replicate this association (OR_s_ = 1.23, *P* = 0.29). Prior significant association for rs3918249 in one Caucasian study (OR = 0.63, *P* = 0.006) was not replicated in meta-analysis of 3 Chinese studies including this study (OR_s_ = 0.91, *P* = 0.13). Significant heterogeneity between non-Chinese and Chinese datasets precluded overall meta-analysis for rs17576 and rs3918249 (*Q* = 0.001 and 0.04 respectively). rs17577 was nominally associated with PACG in one Caucasian study (OR = 1.71, *P* = 0.02), but not in 3 Chinese studies including our study (OR_s_ = 1.20, *P* = 0.07). Overall meta-analysis revealed nominal association for rs17577 and PAC/PACG (OR_s_ = 1.26, *P*_*c*_ = 0.05). Meta-analysis did not show significant association between the other SNPs and PAC/PACG (*P* > 0.47). The largest association study to date did not find significant association between *MMP9* and PAC/PACG in Chinese; meta-analysis with other Chinese datasets did not produce significant association. In most instances combination with non-Chinese datasets was not possible except for one variant showing nominally significant association. More work is needed to define the role of *MMP9* variants in PACG.

## Introduction

Glaucoma is the leading cause of irreversible blindness worldwide, and is characterized by retinal ganglion cell death which results in vision loss [1]. Primary open-angle glaucoma (POAG) and primary angle closure glaucoma (PACG) are two major forms of glaucoma. PACG is characterized by the apposition between the peripheral iris and trabecular meshwork, which causes elevated intraocular pressure (IOP). It is estimated that PACG blinds more people than POAG worldwide [2]. PACG is more prevalent in Asian populations, affecting approximately 0.75% of adult Asians [3]. The prevalence of PACG is estimated to be 1.1% in the Chinese population [3]. PACG is more common than previously thought in Caucasian populations. The prevalence of PACG in those 40 years or more is 0.4% in European derived populations [4]. It is estimated that 581,000 people in the U.S. are affected with PACG today, and this is projected to increase by 18% within the next decade [4].

An unusually higher incidence of PACG among siblings of affected patients than the general population suggests that genetic factors may play an important role in the development of PACG [5,6]. However, the genetic basis of PACG is not well understood. Recently, two genome-wide association studies identified four genetic loci for PACG, including *ABCC5*, *COL11A1*, *PLEKHA7* and an intergenic region between *PCMTD1* and *ST18* on chromosome 8q [7,8]. In addition, candidate gene studies have evaluated common variants in over 50 genes for association with PACG and related phenotypes. A recent systematic review and meta-analysis highlighted the associations of 10 variants in 8 genes/loci with PACG and related phenotypes, including *COL11A1* (rs3753841), *HGF* (rs17427817 and rs5745718), *HSP70* (rs1043618), *MFRP* (rs2510143 and rs3814762), *MMP9* (rs3918249), *NOS3* (rs7830), *PLEKHA7* (rs11024102) and *PCMTD1-ST18* (rs1015213) [9]; however, at least 4 variants of 3 genes in this study (i.e., *MFRP*, *MMP9* and *NOS3*) only showed nominal association and did not survive the Bonferroni correction for multiple testing (all corrected *P* [*P*_*c*_] > 0.05), and thus making the associations of these genes with PACG inconclusive. The equivocal results in this meta-analysis could be due to the relatively small sample sizes and limited statistical power (i.e., only 300–400 cases in total for each variant of the three genes). Further studies with larger samples are required to clarify these associations.

In the present study, to help clarify the association between *matrix metalloproteinase-9* (*MMP9*) and PACG, we evaluated common *MMP9* variants for association with primary angle closure (PAC) and PACG in a large Chinese Han sample. Moreover, we performed a meta-analysis by including this Chinese dataset as well as published results from 5 Chinese studies [10–14] and 2 non-Chinese studies [15,16].

## Methods

### Cases and controls

A total of 1,030 PAC or PACG cases (PAC/PACG) and 499 controls were recruited from the Eye and Ear Nose Throat Hospital, Fudan University. The case group included 572 patients with PACG and 458 patients with PAC. Among cases, 492 patients were acute PAC/PACG, 496 patients were chronic PAC/PACG, and 42 patients were not able to be classified into either acute or chronic PAC/PACG. A complete eye examination was performed for each patient, including examination of the anterior chamber with a slit-lamp, measurement of intraocular pressure (IOP) by Goldmann applanation tonometry, and assessment of fundus photo. Gonioscopy, anterior chamber depth (ACD), axial length (AXL), and visual field were further examined if PAC or PACG was suspected. ACD, AXL and central corneal thickness (CCT) were measured by A-scan ultrasound pachymetry. According to the definitions from the International Society of Geographical and Epidemiological Ophthalmology (ISGEO) [17], PACG was diagnosed based on the presence of glaucomatous optic neuropathy with a vertical cup-disc ratio (VCDR) of 0.7 or greater, peripheral visual loss, an IOP > 21 mmHg, and the presence of at least two quadrants of iridotrabecular contact in which the trabecular meshwork was not visible on gonioscopy. PAC was diagnosed if trabecular obstruction by the peripheral iris had occurred (IOP > 21 mmHg or peripheral anterior synechiae), but without glaucomatous optic neuropathy. Acute PAC/PACG was defined as an episode with (1) a presenting IOP > 28 mmHg; (2) at least two of the following symptoms: ocular or periocular pain, nausea, vomiting, or an antecedent history of intermittent blurring of vision; and (3) at least three of the following signs: conjunctival injection, corneal epithelial edema, mid-dilated nonreactive pupil, or shallow anterior chamber [18]. Chronic PAC/PACG patients consisted of those with no acute signs or symptoms, but met other criteria of PAC/PACG listed above [19]. Patients with secondary angle closure glaucoma due to uveitis, trauma, neovascularization, or any other optic nerve injury affecting either eye were excluded.

Control subjects had no evidence of PAC/PACG based on clinical exam, no family history of glaucoma, no previous surgeries for glaucoma, and no other eye disorders besides senile cataracts.

All the cases and controls were of self-reported Chinese Han ancestry. Demographic and clinical features of the patients with PAC/PACG and controls were shown in **Table 1**.

**Table 1:** Demographic and clinical features of cases and controls in this study.

	PAC	PACG	Acute PAC/PACG	Chronic PAC/PACG	Overall PAC/PACG	Controls
**Number**	458	572	492	496	1030^b^	499
**Female (%)**	74.7	57.3^c^	75.2	53.8^c^	65.1^c^	70.5
**Age (year)**^**a**^	57.9±11.1^d^	57.8±11.8^d^	58.0±11.5^d^	57.9±11.7^d^	57.8±11.5^d^	56.0±12.7
**IOP at diagnosis (mm Hg)**	28.7±15.7	29.3±13.3	31.2±16.7	27.5±11.6	29.0±14.4	N.A.
**Maximum IOP (mm Hg)**	31.4±16.2^d^	33.1±14.1^d^	34.3±17.0^d^	31.1±12.9^d^	32.4±15.1^d^	14.7±2.9
**Vertical cup-disc ratio**	0.43±0.12^d^	0.88±0.09^d^	0.61±0.24^d^	0.80±0.20^d^	0.69±0.24^d^	0.35±0.11
**Anterior chamber depth (mm)**	2.43±0.58	2.55±0.73	2.53±0.84	2.48±0.44	2.50±0.67	N.A.
**Axial length (mm)**	22.1±1.0^d^	22.3±1.0^d^	22.0±1.0^d^	22.4±1.0^d^	22.2±1.0^d^	24.2±1.7
**Center cornea thickness (mm)**	552.2±36.1^d^	536.9±34.0^d^	548.6±37.7^d^	538.8±33.3^d^	543.7±35.8^d^	528.8±33.6

All data were presented as (mean±standard deviation) except gender which was presented as percentage.

Abbreviations: IOP, intraocular pressure; N.A., not available; PAC, primary angle closure; PACG, primary angle closure glaucoma.

^a^ Age at diagnosis for cases and age at enrollment for controls.

^b^ Including 42 patients who were not able to be classified into either acute or chronic PAC/PACG.

^c^ *P* < 0.05 compared to controls according to Chi-squared test.

^d^ *P* < 0.05 compared to controls according to Mann-Whitney U test.

This study followed the tenets of the Declaration of Helsinki and was approved by the ethical committee of Eye and Ear Nose Throat Hospital, Fudan University. Written informed consent was obtained from all patients and controls after explanation of the nature and possible consequences of the study.

### Genotyping

**Fig 1:**
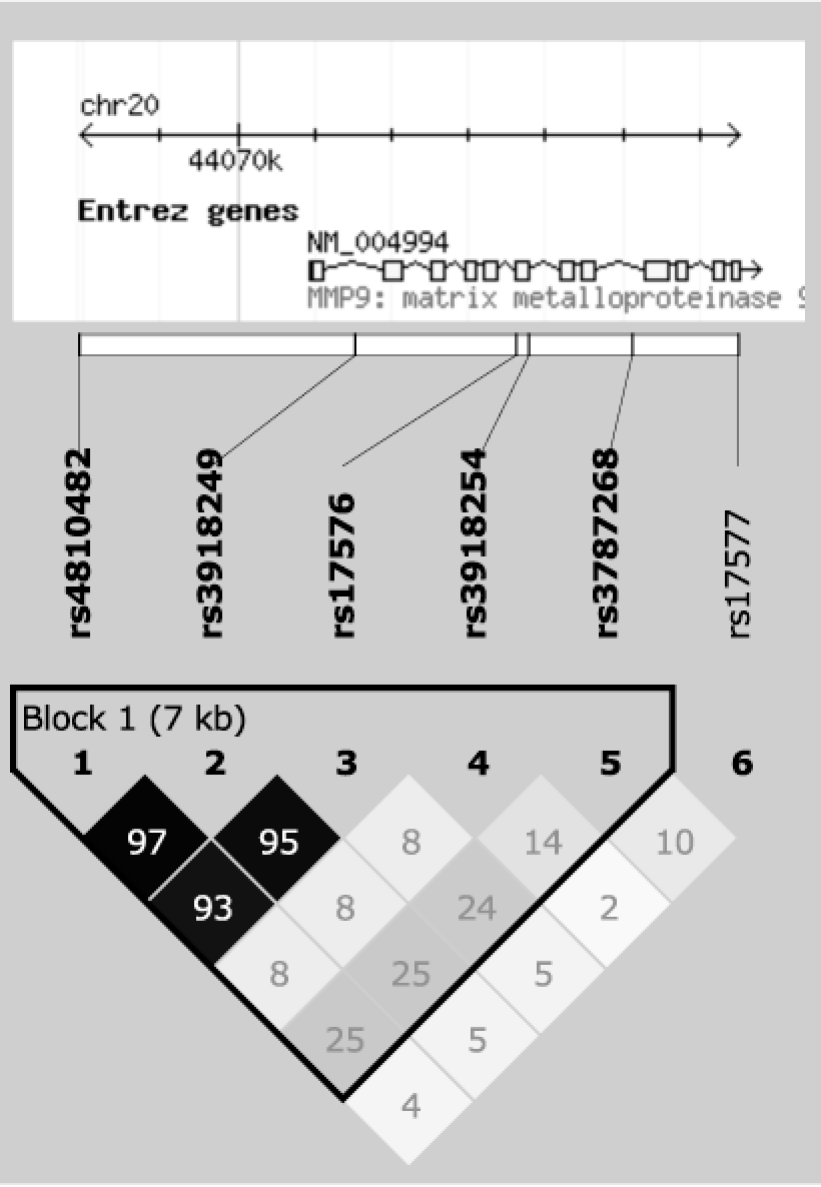
Linkage disequilibrium plot of the 6 tag SNPs around *MMP9* in the Chinese dataset of this study. The numbers in the diamond indicate r^2^; black represented r^2^ = 1, shades of grey indicated 0< r^2^ <1, and white referred to r^2^ = 0. Chromosomal positions were based on NCBI build 36.3 (National Center for Biotechnology Information, Bethesda, MD).

We selected six tag SNPs that captured >95% of alleles at r^2^ greater than 0.8 across the *MMP9* genomic region, including all exons, introns, the 5’- and 3’-UTR, and the 5-kb proximal promoter region (**Fig 1**). Tag SNPs were selected according to the HapMap CHB+JPT data (version 2, release 21) using Haploview (version 4.2) [20]. The minimum minor allele frequency for checking SNPs was set to 0.1. Genotyping was performed by TaqMan assays (Applied Biosystems [ABI], Foster City, CA) according to the manufacturer’s instructions.

### Statistical analysis

The analysis of CCT, ACD and AXL was performed using the average measurements from both eyes. IOP and VCDR were analyzed using the eye with greater values. Chi-squared test was used to compare the difference in sex between cases and controls. Mann-Whitney U test was used to compare the differences in age, IOP, VCDR, ACD, AXL and CCT between cases and controls.

The linkage disequilibrium (LD) plot was generated using Haploview (version 4.2) [20], where squared Pearson correlation coefficient (r^2^) was used to measure LD. Hardy-Weinberg equilibrium was assessed by the chi-squared test. The association analysis was performed using PLINK (version 1.07) [21] for overall PAC/PACG, the subgroups (PAC, PACG, acute PAC/PACG and chronic PAC/PACG), and the subphenotypes (age at diagnosis, IOP at diagnosis, maximum IOP, VCDR, ACD, AXL and CCT). Logistic or linear regression was used to adjust for age and sex in the association analysis. Multiple comparisons were corrected for the number of SNPs for each analysis using the Bonferroni method.

As described in detail previously [22], haplotype analysis was performed with the standard Expectation-Maximization algorithm and the chi-squared test. *P* values were obtained from the haplotype-specific test and the omnibus test. The odds ratio (OR) and 95% confidence interval (CI) were calculated for each of individual haplotypes.

Meta-analysis was performed using the Mantel-Haenszel method, assuming fixed or random effects based on the heterogeneity test results. The heterogeneity between datasets was evaluated using the heterogeneity index (*I*^*2*^) and the Cochran’s *Q* statistic [23]. The forest plot was generated using Inkscape (Release 0.91, The Inkscape Team, 2015, www.inkscape.org) based on the output from the Review Manager software (RevMan, version 5.3; Copenhagen: The Nordic Cochrane Centre, The Cochrane Collaboration, 2014).

Power analysis for association of *MMP9* SNPs with PAC/PACG was performed using the Genetic Power Calculator [24]. The disease prevalence was set as 0.4% for the non-Chinese datasets [4] and 1.1% for the Chinese datasets [3]. The risk allele frequency was set to the same as the marker allele frequency, with 0.38 and 0.34 for the non-Chinese and Chinese datasets based on the allele frequencies of rs17576 in the 1000 Genomes Project [25]. Linkage disequilibrium between the marker and the risk allele was set at D’ = 1.0. The genotypic relative risks for heterozygous (Aa)/high risk homozygous (AA) genotypes were set as 1.78/3.17 and 1.23/1.51 respectively for the non-Chinese and Chinese datasets based on the results of meta-analyses for rs17576, assuming an additive risk model [24].

Power analysis for association of *MMP9* SNPs with subphenotypes in PAC/PACG cases was performed using R scripts [26], assuming a linear model. The phenotypic variance explained by additive effects at the marker of interest was set as 1%-5%.

## Results

All 6 tag SNPs followed Hardy-Weinberg equilibrium in both case and control groups (*P* > 0.1). None of the 6 *MMP9* tag SNPs were significantly associated with overall PAC/PACG (age and sex adjusted *P* > 0.07; **Table 2**) or with the subgroups (age and sex adjusted *P* > 0.03 and 0.08 for PAC and PACG respectively, **Table 2**; age and sex adjusted *P* > 0.04 and 0.08 for acute and chronic PAC/PACG respectively, **S1 Table**; all *P*_*c*_ > 0.18 after correction for multiple testing).

**Table 2:** Single-SNP association analysis of *MMP9* tag SNPs with PACG in this study.

		MAF				PAC		PACG		Overall PAC/PACG	
SNP	MA	PAC	PACG	Overall PAC/PACG	Controls	OR (95%CI)	*P*	OR (95%CI)	*P*	OR (95%CI)	*P*
rs4810482	T	0.27	0.29	0.28	0.31	0.80 (0.65–0.98)	0.03	0.94 (0.78–1.14)	0.51	0.86 (0.73–1.02)	0.11
rs3918249	T	0.27	0.29	0.28	0.31	0.81 (0.66–0.99)	0.04	0.94 (0.78–1.14)	0.53	0.87 (0.74–1.03)	0.14
rs17576	A	0.28	0.30	0.29	0.31	0.85 (0.70–1.04)	0.12	0.96 (0.80–1.16)	0.68	0.90 (0.76–1.06)	0.27
rs3918254	T	0.19	0.18	0.19	0.16	1.24 (0.98–1.57)	0.08	1.16 (0.93–1.45)	0.19	1.21 (0.99–1.48)	0.08
rs3787268	A	0.40	0.38	0.39	0.41	0.96 (0.79–1.15)	0.65	0.86 (0.72–1.02)	0.08	0.90 (0.77–1.05)	0.17
rs17577	A	0.15	0.15	0.15	0.12	1.22 (0.93–1.60)	0.16	1.23 (0.95–1.59)	0.11	1.23 (0.98–1.55)	0.07

Abbreviation: MA, minor allele; MAF, minor allele frequency; PAC, primary angle closure; PACG, primary angle closure glaucoma. The Bonferroni corrected significance level was set as 0.008 (0.05/6).

Haplotype analysis of 6 tag SNPs did not find significant association of *MMP9* with overall PAC/PACG (*P* > 0.11; **S2 Table**), or the subgroups (*P* > 0.05 and 0.16 for PAC and PACG respectively, **S2 Table**; *P* > 0.09 and 0.08 for acute and chronic PAC/PACG respectively; **S3 Table**).

Analysis of subphenotypes in the PAC/PACG cases did not reveal significant association of *MMP9* SNPs with age at diagnosis, IOP at diagnosis, maximum IOP, VCDR, ACD, AXL and CCT (age and sex adjusted *P* > 0.15; **S4** and **S5 Tables**).

**Fig 2:**
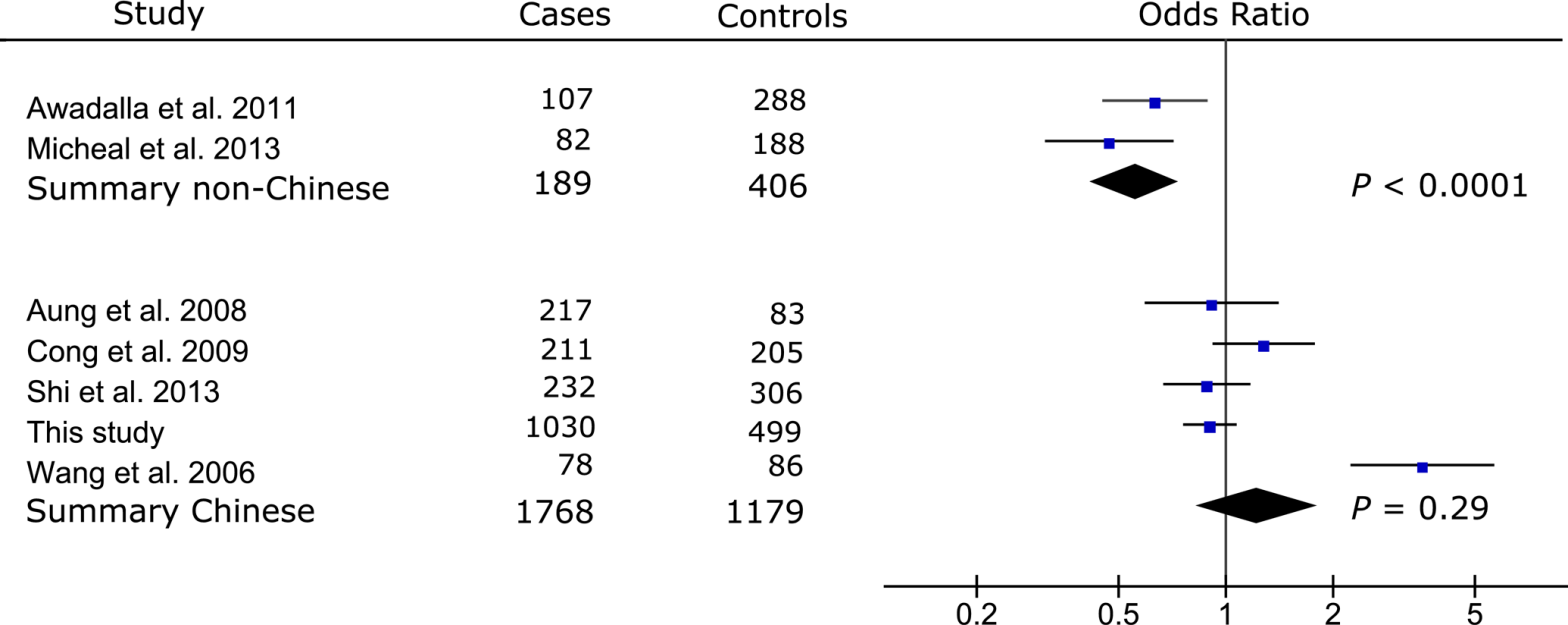
Meta-analysis with prior studies of the association between rs17576 and PAC/PACG. Odds ratio was calculated per each increase in minor allele A. The summary odds ratio was 0.56 (95% CI: 0.42–0.74) for the non-Chinese populations and 1.23 (95% CI: 0.83–1.82) for the Chinese populations. Significant heterogeneity between the non-Chinese and Chinese populations precluded an overall meta-analysis for rs17576 (*Q* = 0.001, *I*^*2*^ = 90%). The Bonferroni corrected significance level was set as 0.01 (0.05/5).

**Fig 3:**
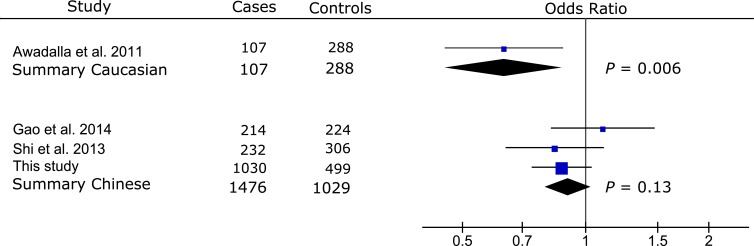
Meta-analysis with prior studies of the association between rs3918249 and PAC/PACG. Odds ratio was calculated per each increase in minor allele T. The summary odds ratio was 0.63 (95% CI: 0.45–0.88) for the Caucasian population and 0.91 (95% CI: 0.80–1.03) for the Chinese populations. Significant heterogeneity between the Caucasian and Chinese populations precluded an overall meta-analysis for rs3918249 (*Q* = 0.04, *I*^*2*^ = 75%). The Bonferroni corrected significance level was set as 0.01 (0.05/5).

**Fig 4:**
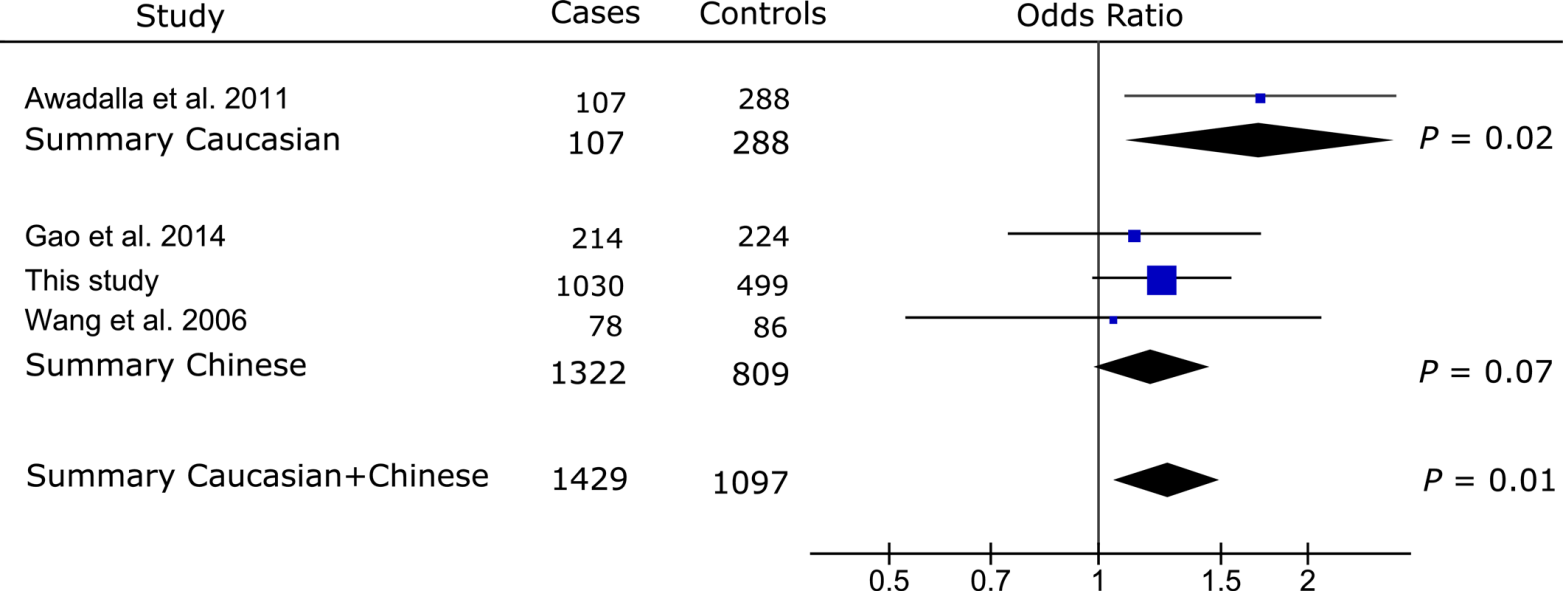
Meta-analysis with prior studies of the association between rs17577 and PAC/PACG. Odds ratio was calculated per each increase in minor allele A. The summary odds ratio was 1.71 (95% CI: 1.09–2.67) for the Caucasian population, 1.20 (95% CI: 0.99–1.45) for the Chinese populations, and 1.26 (95%CI: 1.06–1.50) for the combined Caucasian + Chinese populations, respectively. The odds ratios between the Caucasian and Chinese datasets were not significantly heterogeneous (*Q* = 0.15, *I*^*2*^ = 52%). The Bonferroni corrected significance level was set as 0.01 (0.05/5).

Meta-analysis of the published data from two non-Chinese populations [15,16] showed significant association between rs17576 and PACG (summary OR [OR_s_] = 0.56, 95% CI: 0.42–0.74, *P* < 0.0001; **Fig 2**). Meta-analysis of our Chinese data with the published data from 4 Chinese populations [10–13] did not find significant association of rs17576 with PAC/PACG (OR_s_ = 1.23, 95% CI: 0.83–1.82, *P* = 0.29; **Fig 2**). Significant heterogeneity between the non-Chinese and Chinese populations precluded an overall meta-analysis for rs17576 (*Q* = 0.001, *I*^*2*^ = 90%; **Fig 2**). Significant association was reported for rs3918249 in one study of the Caucasian population (OR = 0.63, 95% CI: 0.45–0.88, *P* = 0.006; **Fig 3**), while no significant association was found for rs3918249 in the meta-analysis of 3 Chinese studies that included this work (OR_s_ = 0.91, 95%CI: 0.80–1.03, *P* = 0.13; **Fig 3**). Significant heterogeneity was observed between the Caucasian and Chinese studies (*Q* = 0.04, *I*^*2*^ = 75%; **Fig 3**), making it impossible to include the Caucasian data in the meta-analysis of rs3918249. Similarly, rs17577 was nominally associated with PACG in one Caucasian study (OR = 1.71, 95% CI: 1.09–2.67, *P* = 0.02; **Fig 4**), but not in three Chinese studies that included our data (OR_s_ = 1.20, 95% CI: 0.99–1.45, *P* = 0.07; **Fig 4**). Since no significant heterogeneity was found between the Caucasian and Chinese studies (*Q* = 0.15, *I*^*2*^ = 52%; **Fig 4**), an overall meta-analysis of the combined Caucasian and Chinese populations showed nominal association between rs17577 and PAC/PACG (OR_s_ = 1.26, 95%CI: 1.06–1.50, *P* = 0.01, *P*_*c*_ = 0.05; **Fig 4**). Meta-analysis did not show significant association between rs3918254 and PAC/PACG (OR_s_ = 1.01, 95%CI: 0.68–1.50, *P* = 0.95; **S1 Fig**) or between rs3787268 and PAC/PACG (OR_s_ = 0.95, 95%CI: 0.84–1.08, *P* = 0.47; **S2 Fig**).

## Discussion

In the present study, we evaluated tag SNPs that captured >95% of genetic variation in the *MMP9* gene region as well as the proximal 5-kb promoter for association with PACG in a large Chinese case-control dataset. No significant association was found between common SNPs or haplotypes of *MMP9* and PAC/PACG in this Chinese sample (**Table 2, S2 Table**). MMP9 is a member of a large family of endopeptidases which are involved in the remodeling and degradation of extracellular matrix (ECM) [27]. Functional variants have been identified in the *MMP9* gene. It is hypothesized that shorter axial length observed in patients with PACG might be due to a change in the MMP9 activity resulting in ECM remodeling during eye growth and development [10].

A coding variant rs17576 in exon 6 of *MMP9* leads to the substitution of arginine by glutamine, which might change the enzyme activity of MMP9 [28]. Previous studies have identified significant associations between rs17576 and PACG in one Australian Caucasian population [15], one Pakistani population [16] and one Chinese population [10]. However, three subsequent studies on Chinese patients did not replicate this association [11–13]. Our study also failed to find an association between rs17576 and PAC/PACG in a larger Chinese sample (**Table 2, S2 Table**). To further evaluate this association, we performed a meta-analysis using our genotype data for the Chinese dataset as well as published data for the Chinese and non-Chinese datasets. Our meta-analysis revealed significant association between rs17576 and PACG in the non-Chinese populations but not in the Chinese populations (**Fig 2**). Significant heterogeneity between the non-Chinese and Chinese datasets was observed for rs17576 precluding an overall meta-analysis for this SNP (**Fig 2**). Notably, the risk allele is “G” in the non-Chinese populations, which is different from that in the Chinese populations (i.e., “A”). Such “flip-flop” associations may be due to population-specific effects [29], which have been well documented in genetic association studies such as the association of *LOXL1* with exfoliation syndrome [30–32]. Alternatively, the limited sample sizes in the non-Chinese populations might have biased the observed associations between rs17576 and PACG. Further analyses using larger samples or other populations such as Africans will be helpful to clarify this issue.

The findings of significant association between common *MMP9* variants and PACG in the non-Chinese populations but not in the Chinese populations were further supported by the results of additional two SNPs in *MMP9*, rs3918249 (**Fig 3)** and rs17577 (**Fig 4**). Both rs3918249 and rs17577 were reported to be significantly associated with PACG in a Caucasian study [15]. However, meta-analyses of our data with the published data from the Chinese populations [10,13,14] did not identify significant association of rs3918249 and rs17577 with PAC/PACG (**Figs 3** and **4**). An overall meta-analysis of the combined Caucasian and Chinese populations revealed nominal association for rs17577 (*P*_*c*_ = 0.05; **Fig 4**). Our meta-analysis of the combined Caucasian and Chinese datasets did not find significant association of rs3918254 and rs3787268 with PAC/PACG (**S1** and **S2 Figs**), suggesting that these SNPs are not likely to contribute to the disease. Alternatively, the relatively low frequency of the risk allele for rs3918254 might have prevented us from detecting the association due to insufficient statistical power, particularly in the Caucasian population.

Polymorphisms in the promoter region of *MMP9* have been reported to affect the gene transcription [16]. The promoter region has been poorly investigated in previous association studies for *MMP9* and PACG. Only one SNP, rs3918242, located at 1,562 base pairs upstream of the *MMP9* start codon, was investigated in the Pakistani study [16], while promoter region SNPs were not included in other studies [10–15].The present study is the first time that an association between the *MMP9* promoter region and PAC/PACG has been evaluated using tag SNPs. We did not identify significant association between the *MMP9* promoter region and PAC/PACG in the Chinese dataset (**Table 2**), consistent with the results from the Pakistani study [16]. These results suggest that the *MMP9* promoter may not contribute to PACG risk, although further studies using larger datasets are required to confirm these findings.

A major limitation of previous *MMP9* association studies is the relatively small sample sizes (no more than 300 in either case or control group). In this study, we enrolled a larger sample (1,030 cases and 499 controls), making it the largest study on *MMP9* common variants and PAC/PACG to date. Our meta-analysis of rs17576 suggests that genetic effects of *MMP9* variants are likely to be modest and on the order of 1.78/3.17 and 1.23/1.51 for Aa/AA in the non-Chinese and Chinese datasets respectively (**Fig 2**). We estimated that we had 98% of power in all the non-Chinese datasets (189 cases and 406 controls), 48% of power in our Chinese dataset (1,030 cases and 499 controls), and 88% of power in all the Chinese datasets (1,768 cases and 1,179 controls) to detect this modest genetic effect [24]. Notably, the GWAS in the Chinese populations by Vithana et al. (1,854 cases and 9,608 controls in the discovery dataset) had only 63% power to detect the association between rs17576 and PACG, and this may explain why this study did not identify significant association [8].

Our analysis of subphenotypes in the PAC/PACG cases did not find significant association of *MMP9* variants with age at diagnosis, IOP at diagnosis, maximum IOP, VCDR, ACD, AXL and CCT (**S4** and **S5 Tables**), suggesting that common variants in *MMP9* might not contribute to the disease severity. We estimated that we had 71.6%-99.9% of power to detect the genetic effect of *MMP9* SNPs on these quantitative subphenotypes, assuming the phenotypic variance explained by additive effects at the marker of interest as 1%-5%.

## Conclusions

We have evaluated common variants in *MMP9* as genetic risk factors for PAC/PACG. This is the largest association study on *MMP9* and PAC/PACG to date. Prior results suggest that common *MMP9* variants may contribute to the modest risk for PACG in the non-Chinese population but our analysis suggests this not the case in the Chinese population. Additional studies using larger datasets will be necessary to confirm these population-specific findings.

## Supporting Information

S1 Fig Meta-analysis with prior studies of the association between rs3918254 and PAC/PACG. Odds ratio was calculated per each increase in minor allele A. The summary odds ratio was 2.68 (95% CI: 0.17–43.00) for the Caucasian population, 0.99 (95% CI: 0.65–1.52) for the Chinese populations, and 1.01 (95%CI: 0.68–1.50) for the combined Caucasian + Chinese populations, respectively. The odds ratios between the Caucasian and Chinese datasets were not significantly heterogeneous (*Q* = 0.49, *I*^*2*^ = 0%). The Bonferroni corrected significance level was set as 0.01 (0.05/5).
(DOCX)

S2 Fig Meta-analysis with prior studies of the association between rs3787268 and PAC/PACG. Odds ratio was calculated per each increase in minor allele A. The summary odds ratio was 1.26 (95% CI: 0.86–1.86) for the Caucasian population, 0.92 (95% CI: 0.81–1.06) for the Chinese populations, and 0.95 (95%CI: 0.84–1.08) for the combined Caucasian + Chinese populations, respectively. The odds ratios between the Caucasian and Chinese datasets were not significantly heterogeneous (*Q* = 0.13, *I*^*2*^ = 56%). The Bonferroni corrected significance level was set as 0.01 (0.05/5).
(DOCX)

S1 Table Single-SNP association analysis of *MMP9* tag SNPs with acute and chronic PAC/PACG in this study. (DOCX)

S2 Table Haplotype association analysis of *MMP9* tag SNPs with PAC/PACG in this study. (DOCX)

S3 Table Haplotype association analysis of *MMP9* tag SNPs with acute and chronic PAC/PACG in this study. (DOCX)

S4 Table Association analysis of *MMP9* tag SNPs with age at diagnosis, IOP and VCDR in PAC/PACG cases of this study. (DOCX)

S5 Table Association analysis of *MMP9* tag SNPs with ACD, AXL and CCT in PAC/PACG cases of this study. (DOCX)
