# Supplementary material for: Association of Matrix Metalloproteinase-9 (MMP9) Variants with Primary Angle Closure and Primary Angle Closure Glaucoma
Source: PLoS One. 2016 Jun 7;11(6):e0157093. doi: 10.1371/journal.pone.0157093 (PMC4896618; doi:10.1371/journal.pone.0157093)
Supplement: S1 Fig — Odds ratio was calculated per each increase in minor allele A. The summary odds ratio was 2.68 (95% CI: 0.17–43.00) for the Caucasian population, 0.99 (95% CI: 0.65–1.52) for the Chinese populations, and 1.01 (95%CI: 0.68–1.50) for the combined Caucasian + Chinese populations, respectively. The odds ratios between the Caucasian and Chinese datasets were not significantly heterogeneous (Q = 0.49, I2 = 0%). The Bonferroni corrected significance level was set as 0.01 (0.05/5). (DOCX) [file pone.0157093.s001.docx]

**
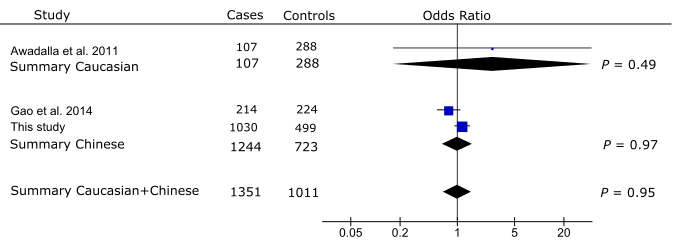
**

**S1 Fig.** **Meta-analysis with prior studies of the association between rs3918254 and PAC/PACG.** Odds ratio was calculated per each increase in minor allele A. The summary odds ratio was 2.68 (95% CI: 0.17-43.00) for the Caucasian population, 0.99 (95% CI: 0.65-1.52) for the Chinese populations, and 1.01 (95%CI: 0.68-1.50) for the combined Caucasian + Chinese populations, respectively. The odds ratios between the Caucasian and Chinese datasets were not significantly heterogeneous (*Q* = 0.49, *I^2^* = 0%). The Bonferroni corrected significance level was set as 0.01 (0.05/5).
