# Supplementary material for: Association of Matrix Metalloproteinase-9 (MMP9) Variants with Primary Angle Closure and Primary Angle Closure Glaucoma
Source: PLoS One. 2016 Jun 7;11(6):e0157093. doi: 10.1371/journal.pone.0157093 (PMC4896618; doi:10.1371/journal.pone.0157093)
Supplement: S2 Fig — Odds ratio was calculated per each increase in minor allele A. The summary odds ratio was 1.26 (95% CI: 0.86–1.86) for the Caucasian population, 0.92 (95% CI: 0.81–1.06) for the Chinese populations, and 0.95 (95%CI: 0.84–1.08) for the combined Caucasian + Chinese populations, respectively. The odds ratios between the Caucasian and Chinese datasets were not significantly heterogeneous (Q = 0.13, I2 = 56%). The Bonferroni corrected significance level was set as 0.01 (0.05/5). (DOCX) [file pone.0157093.s002.docx]

**
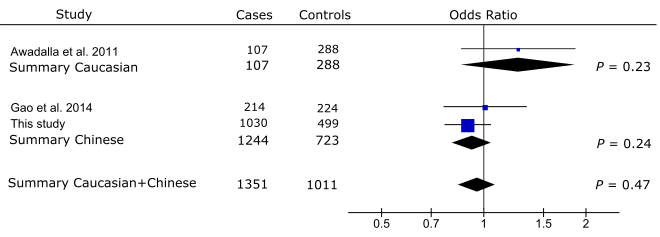
**

**S2 Fig.** **Meta-analysis with prior studies of the association between rs3787268 and PAC/PACG.** Odds ratio was calculated per each increase in minor allele A. The summary odds ratio was 1.26 (95% CI: 0.86-1.86) for the Caucasian population, 0.92 (95% CI: 0.81-1.06) for the Chinese populations, and 0.95 (95%CI: 0.84-1.08) for the combined Caucasian + Chinese populations, respectively. The odds ratios between the Caucasian and Chinese datasets were not significantly heterogeneous (*Q* = 0.13, *I^2^* = 56%). The Bonferroni corrected significance level was set as 0.01 (0.05/5).
