# Supplementary material for: Association of Matrix Metalloproteinase-9 (MMP9) Variants with Primary Angle Closure and Primary Angle Closure Glaucoma
Source: PLoS One. 2016 Jun 7;11(6):e0157093. doi: 10.1371/journal.pone.0157093 (PMC4896618; doi:10.1371/journal.pone.0157093)
Supplement: S1 Table — (DOCX) [file pone.0157093.s003.docx]

**S1 Table. Single-SNP association analysis of *MMP9* tag SNPs with** **acute and chronic PAC/PACG in this study**

|  |  | **MAF** | | |  | **Acute PAC/PACG** |  |  | **Chronic PAC/PACG** |  |
| --- | --- | --- | --- | --- | --- | --- | --- | --- | --- | --- |
| **SNP** | **MA** | **Acute PAC/PACG** | **Chronic PAC/PACG** | **Controls** |  | **OR (95%CI)** | ***P*** |  | **OR (95%CI)** | ***P*** |
| rs4810482 | T | 0.27 | 0.29 | 0.31 |  | 0.83 (0.68-1.00) | 0.04 |  | 0.89 (0.74-1.08) | 0.39 |
| rs3918249 | T | 0.27 | 0.29 | 0.31 |  | 0.83 (0.68-1.01) | 0.05 |  | 0.90 (0.74-1.10) | 0.42 |
| rs17576 | A | 0.28 | 0.29 | 0.31 |  | 0.86 (0.71-1.05) | 0.11 |  | 0.92 (0.76-1.12) | 0.58 |
| rs3918254 | T | 0.18 | 0.19 | 0.16 |  | 1.15 (0.91-1.45) | 0.24 |  | 1.26 (1.00-1.59) | 0.08 |
| rs3787268 | A | 0.40 | 0.38 | 0.41 |  | 0.93 (0.78-1.12) | 0.47 |  | 0.88 (0.73-1.05) | 0.18 |
| rs17577 | A | 0.15 | 0.14 | 0.12 |  | 1.31 (1.01-1.69) | 0.04 |  | 1.17 (0.90-1.52) | 0.31 |

Abbreviation: MA, minor allele; MAF, minor allele frequency; PAC, primary angle closure; PACG, primary angle closure glaucoma.

The Bonferroni corrected significance level was set as 0.008 (0.05/6).
