# Supplementary material for: Association of Matrix Metalloproteinase-9 (MMP9) Variants with Primary Angle Closure and Primary Angle Closure Glaucoma
Source: PLoS One. 2016 Jun 7;11(6):e0157093. doi: 10.1371/journal.pone.0157093 (PMC4896618; doi:10.1371/journal.pone.0157093)
Supplement: S2 Table — (DOCX) [file pone.0157093.s004.docx]

**S2 Table. Haplotype association analysis of *MMP9* tag SNPs with PAC/PACG in this study**

|  | **Haplotype frequency** | | | |  | **PAC** | |  | **PACG** | |  | **Overall PAC/PACG** | |
| --- | --- | --- | --- | --- | --- | --- | --- | --- | --- | --- | --- | --- | --- |
| **Haplotype^a^** | **PAC** | **PACG** | **Overall PAC/PACG** | **Controls** |  | **OR (95%CI)^b^** | ***P*^c^** |  | **OR (95%CI)^b^** | ***P*^c^** |  | **OR (95%CI)^b^** | ***P*^c^** |
| CCGCGA | 0.14 | 0.14 | 0.14 | 0.12 |  | 1.17 (0.89-1.53) | 0.28 |  | 1.20 (0.93-1.54) | 0.18 |  | 1.18 (0.94-1.48) | 0.16 |
| CCGCAG | 0.41 | 0.38 | 0.39 | 0.41 |  | 0.99 (0.83-1.19) | 0.94 |  | 0.88 (0.74-1.05) | 0.16 |  | 0.93 (0.80-1.09) | 0.36 |
| CCGTGG | 0.19 | 0.19 | 0.19 | 0.16 |  | 1.19 (0.94-1.51) | 0.14 |  | 1.17 (0.93-1.46) | 0.17 |  | 1.18 (0.97-1.45) | 0.11 |
| TTACGG | 0.26 | 0.29 | 0.28 | 0.31 |  | 0.82 (0.67-1.00) | 0.05 |  | 0.94 (0.78-1..13) | 0.53 |  | 0.88 (0.75-1.04) | 0.16 |
| Total | 1.00 | 1.00 | 1.00 | 1.00 |  |  | 0.14^d^ |  |  | 0.21^d^ |  |  | 0.12^d^ |

Abbreviation: PAC, primary angle closure; PACG, primary angle closure glaucoma.

^a^ Haplotypes were constructed in the order of rs4810482|rs3918249|rs17576|rs3918254|rs3787268|rs17577.

^b^ OR and 95%CI were calculated for each of individual haplotypes compared to all the other haplotypes.

^c^ Obtained from the haplotype-specific test using PLINK. The Bonferroni corrected significance level was set as 0.0125 (0.05/4).

^d^ Obtained from the omnibus test using PLINK.
