# Supplementary material for: Association of Matrix Metalloproteinase-9 (MMP9) Variants with Primary Angle Closure and Primary Angle Closure Glaucoma
Source: PLoS One. 2016 Jun 7;11(6):e0157093. doi: 10.1371/journal.pone.0157093 (PMC4896618; doi:10.1371/journal.pone.0157093)
Supplement: S3 Table — (DOCX) [file pone.0157093.s005.docx]

**S3 Table. Haplotype association analysis of *MMP9* tag SNPs with acute and chronic PAC/PACG in this study**

|  | **Haplotype frequency** | | |  | **Acute PAC/PACG** | |  | **Chronic PAC/PACG** | |
| --- | --- | --- | --- | --- | --- | --- | --- | --- | --- |
| **Haplotype^a^** | **Acute PAC/PACG** | **Chronic PAC/PACG** | **Controls** |  | **OR (95%CI)^b^** | ***P*^c^** |  | **OR (95%CI)^b^** | ***P*^c^** |
| CCGCGA | 0.15 | 0.13 | 0.12 |  | 1.26 (0.98-1.64) | 0.09 |  | 1.13 (0.87-1.48) | 0.37 |
| CCGCAG | 0.40 | 0.39 | 0.41 |  | 0.97 (0.81-1.16) | 0.73 |  | 0.90 (0.75-1.08) | 0.26 |
| CCGTGG | 0.18 | 0.19 | 0.16 |  | 1.12 (0.88-1.41) | 0.35 |  | 1.23 (0.98-1.55) | 0.08 |
| TTACGG | 0.27 | 0.29 | 0.31 |  | 0.84 (0.69-1.02) | 0.09 |  | 0.91 (0.75-1.11) | 0.35 |
| Total | 1.00 | 1.00 | 1.00 |  |  | 0.15^d^ |  |  | 0.20^d^ |

Abbreviation: PAC, primary angle closure; PACG, primary angle closure glaucoma.

^a^ Haplotypes were constructed in the order of rs4810482|rs3918249|rs17576|rs3918254|rs3787268|rs17577.

^b^ OR and 95%CI were calculated for each of individual haplotypes compared to all the other haplotypes.

^c^ Obtained from the haplotype-specific test using PLINK. The Bonferroni corrected significance level was set as 0.0125 (0.05/4).

^d^ Obtained from the omnibus test using PLINK.
