# Supplementary material for: Association of Matrix Metalloproteinase-9 (MMP9) Variants with Primary Angle Closure and Primary Angle Closure Glaucoma
Source: PLoS One. 2016 Jun 7;11(6):e0157093. doi: 10.1371/journal.pone.0157093 (PMC4896618; doi:10.1371/journal.pone.0157093)
Supplement: S4 Table — (DOCX) [file pone.0157093.s006.docx]

**S4 Table. Association analysis of *MMP9* tag SNPs with age at diagnosis, IOP and VCDR in PAC/PACG cases of this study**

|  |  |  | **Age_dx** |  |  |  | **IOP_dx** |  |  |  | **IOP_max** |  |  |  | **VCDR** |  |
| --- | --- | --- | --- | --- | --- | --- | --- | --- | --- | --- | --- | --- | --- | --- | --- | --- |
| **SNP** | **MA** | ***β*** | ***S.E.*** | ***P*** |  | ***β*** | ***S.E.*** | ***P*** |  | ***β*** | ***S.E.*** | ***P*** |  | ***β*** | ***S.E.*** | ***P*** |
| rs4810482 | T | -0.25 | 0.57 | 0.66 |  | -0.38 | 0.73 | 0.60 |  | -0.76 | 0.76 | 0.32 |  | 0.008 | 0.01 | 0.49 |
| rs3918249 | T | -0.39 | 0.57 | 0.49 |  | -0.67 | 0.73 | 0.36 |  | -0.89 | 0.76 | 0.24 |  | 0.005 | 0.01 | 0.65 |
| rs17576 | A | -0.51 | 0.56 | 0.36 |  | -0.74 | 0.71 | 0.30 |  | -0.88 | 0.74 | 0.24 |  | 0.002 | 0.01 | 0.84 |
| rs3918254 | T | -0.24 | 0.64 | 0.71 |  | -0.34 | 0.82 | 0.68 |  | 0.52 | 0.86 | 0.54 |  | -0.006 | 0.01 | 0.66 |
| rs3787268 | A | 0.76 | 0.53 | 0.15 |  | 0.86 | 0.68 | 0.21 |  | 0.46 | 0.71 | 0.52 |  | -0.007 | 0.01 | 0.50 |
| rs17577 | A | -0.51 | 0.72 | 0.48 |  | -0.50 | 0.93 | 0.59 |  | -0.28 | 0.96 | 0.77 |  | 0.002 | 0.02 | 0.91 |

Abbreviation: Age_dx, age at diagnosis; IOP_dx, intraocular pressure at diagnosis; IOP_max, maximum intraocular pressure; MA, minor allele; PAC, primary angle closure; PACG, primary angle closure glaucoma; *S.E.*, standard error; VCDR, vertical cup-disc ratio.

The Bonferroni corrected significance level was set as 0.008 (0.05/6).
