# Supplementary material for: Association of Matrix Metalloproteinase-9 (MMP9) Variants with Primary Angle Closure and Primary Angle Closure Glaucoma
Source: PLoS One. 2016 Jun 7;11(6):e0157093. doi: 10.1371/journal.pone.0157093 (PMC4896618; doi:10.1371/journal.pone.0157093)
Supplement: S5 Table — (DOCX) [file pone.0157093.s007.docx]

**S5 Table. Association analysis of *MMP9* tag SNPs with ACD, AXL and CCT in PAC/PACG cases of this study**

|  |  |  | **ACD** |  |  |  | **AXL** |  |  |  | **CCT** |  |
| --- | --- | --- | --- | --- | --- | --- | --- | --- | --- | --- | --- | --- |
| **SNP** | **MA** | ***β*** | ***S.E.*** | ***P*** |  | ***β*** | ***S.E.*** | ***P*** |  | ***β*** | ***S.E.*** | ***P*** |
| rs4810482 | T | -0.01 | 0.04 | 0.79 |  | 0.02 | 0.05 | 0.66 |  | 0.90 | 1.94 | 0.64 |
| rs3918249 | T | -0.01 | 0.04 | 0.76 |  | 0.03 | 0.05 | 0.60 |  | 1.01 | 1.94 | 0.60 |
| rs17576 | A | -0.03 | 0.04 | 0.51 |  | 0.02 | 0.05 | 0.67 |  | 0.36 | 1.90 | 0.85 |
| rs3918254 | T | 0.003 | 0.05 | 0.95 |  | -0.02 | 0.05 | 0.77 |  | -0.32 | 2.18 | 0.88 |
| rs3787268 | A | 0.04 | 0.04 | 0.33 |  | -0.01 | 0.05 | 0.80 |  | 0.73 | 1.83 | 0.69 |
| rs17577 | A | -0.04 | 0.05 | 0.41 |  | 0.02 | 0.06 | 0.73 |  | -2.92 | 2.39 | 0.22 |

Abbreviation: ACD, anterior chamber depth; AXL, axial length; CCT, center cornea thickness; MA, minor allele; PAC, primary angle closure; PACG, primary angle closure glaucoma; *S.E.*, standard error.

The Bonferroni corrected significance level was set as 0.008 (0.05/6).
